# Seeing the Invisible: Revealing Atrial Ablation Lesions Using Hyperspectral Imaging Approach

**DOI:** 10.1371/journal.pone.0167760

**Published:** 2016-12-08

**Authors:** Narine Muselimyan, Luther M. Swift, Huda Asfour, Tigran Chahbazian, Ramesh Mazhari, Marco A. Mercader, Narine A. Sarvazyan

**Affiliations:** 1 Department of Pharmacology and Physiology, The George Washington University School of Medicine and Health Sciences, Washington, District of Columbia, United States of America; 2 Strasbourg Medical University, Strasbourg, France; 3 Division of Cardiology, The George Washington University, Medical Faculty Associates, Washington, District of Columbia, United States of America; University of Minnesota, UNITED STATES

## Abstract

**Background:**

Currently, there are limited means for high-resolution monitoring of tissue injury during radiofrequency ablation procedures.

**Objective:**

To develop the next generation of visualization catheters that can reveal irreversible atrial muscle damage caused by ablation and identify viability gaps between the lesions.

**Methods:**

Radiofrequency lesions were placed on the endocardial surfaces of excised human and bovine atria and left ventricles of blood perfused rat hearts. Tissue was illuminated with 365nm light and a series of images were acquired from individual spectral bands within 420-720nm range. By extracting spectral profiles of individual pixels and spectral unmixing, the relative contribution of ablated and unablated spectra to each pixel was then displayed. Results of spectral unmixing were compared to lesion pathology.

**Results:**

RF ablation caused significant changes in the tissue autofluorescence profile. The magnitude of these spectral changes in human left atrium was relatively small (< 10% of peak fluorescence value), yet highly significant. Spectral unmixing of hyperspectral datasets enabled high spatial resolution, in-situ delineation of radiofrequency lesion boundaries without the need for exogenous markers. Lesion dimensions derived from hyperspectral imaging approach strongly correlated with histological outcomes. Presence of blood within the myocardium decreased the amplitude of the autofluorescence spectra while having minimal effect on their overall shapes. As a result, the ability of hyperspectral imaging to delineate ablation lesions in vivo was not affected.

**Conclusions:**

Hyperspectral imaging greatly increases the contrast between ablated and unablated tissue enabling visualization of viability gaps at clinically relevant locations. Data supports the possibility for developing percutaneous hyperspectral catheters for high-resolution ablation guidance.

## Introduction

Atrial fibrillation (AF) remains one of the most significant health burdens and is expected to affect over 12 million people in the United States by 2050[1]. One of the main treatment options to cure AF is to ablate abnormal sources of electrical activity using percutaneous radiofrequency (RF) catheters. However, the lack of high-resolution, real-time surgical guidance technologies for RF ablation procedures often leads to high rates of AF recurrence[2].

The endocardial surface of human left atrium is covered by thick interwoven layers of collagen and elastin. This yields a highly autofluorescent and reflective endocardial surface, obscuring RF-induced damage to the muscle below. We hypothesized that small spectral differences between ablated and unablated tissue would be nevertheless sufficient to reliably identify underlying muscle damage. To acquire and analyze these small spectral differences, we employed a powerful optical modality called hyperspectal imaging (HSI). HSI captures the spectrum of each pixel in an image, followed by post-acquisition analysis to classify pixels based on their spectral signatures.

HSI was initially developed for aerial or satellite-based remote sensing, but is rapidly gaining recognition for a variety of biomedical applications including cancer detection, drug delivery, or tissue oxygenation[3–6]. We have recently shown that HSI can help outline ablation lesions in cardiac tissues of young pigs[7]. Encouraged by these animal data we proceeded to demonstrate the feasibility of using autofluorescence HSI to visualize RF ablation lesions in human atrial tissue from a clinically relevant age group.

## Methods

### Ablation procedures

The protocol to obtain donated human tissue was approved by the Organ and Tissue Advisory Committee, the Board of Directors of the Washington Regional Transplant Community (WRTC, Washington, DC), the George Washington University and the Inova Fairfax Hospital Institutional Review Boards. Explanted hearts were cardioplegically arrested, cooled to +4°C in the operating room following aortic cross-clamp and transported to the laboratory on ice within 3-4h after excision. RF energy was delivered with either a non-irrigated (EP Technologies, Boston Scientific) or open irrigated ablation catheter (Nocturnal Product Development LLC, Cary, NC). A 4mm tip was placed perpendicular to the endocardial surface, and ablation durations varied from 5 to 30 seconds with tip temperatures ranging between 50 to 70°C.

### Animal studies

Freshly excised bovine atrial tissue was obtained from a local abattoir (Silver Ridge Farm, 73 Silver Ridge Ln, Fredericksburg, VA 22405). Bovine hearts were placed on ice immediately after the excision and delivered to the lab within 1-2h. *In vivo* ablations were performed in Sprague-Dawley rats (200-300g). Animals were anesthetized with an intraperitoneal injection cocktail of ketamine/xylaxine (75mg/kg and 5mg/kg respectively). Upon cessation of any pain reflex from foot and tail pinch, the animal was placed in an ice bath to slow the heart rate, and the chest was opened to expose the heart surface. An ablation was placed on the surface of the left ventricle and imaged immediately. The heart was then excised for further imaging. The procedure follows the current American Veterinary Medical Association guidelines on anesthesia and euthanasia. For blood-free imaging, the heart was removed, and perfused with saline followed by HSI acquisition. All animal experiments were conducted in full accordance with the approved George Washington University School of Medicine and Health Sciences IACUC protocol #243.

### Hyperspectral imaging approach and protocol

To compose a three dimensional HSI hypercube with three axes (x,y,λ), an object can be imaged in a number of ways: by either moving the sample across a linear spectral detector (HSI modality called pushbroom-HSI), by altering excitation wavelength (HSI modality called source-HSI), or by using a bandpass or a tunable filter in front of the camera while the illumination source stays the same. The latter modality can be referred to as detector-based HSI and is the method used in this study. Samples were illuminated with a fixed 365nm light source (LED spotlight, Mightex, Pleasanton, CA), while a liquid tunable filter in front of a CCD camera was sequentially tuned from 420nm to 720nm in 10nm steps using Nuance FX hyperspectral imaging system hardware and software (PerkinElmer/Cri, Waltham, MA). A Nikon Micro-Nikkor 60mm f/2.8D lens in front of the camera included a pair of aspherical elements to avoid spatial aberrations and an extra-low-dispersion glass element to minimize chromatic aberrations. A 4x3cm field-of-view captured at 1392x1040 pixels resulted in ~30 micron/pixel spatial resolution. After each hyperstack was acquired, Nuance FX analysis software was used to perform linear unmixing in order to derive greyscale HSI component images[8]. To provide reference spectra for linear unmixing of HSI hyperstacks, a user placed ~10x10 pixel region-of-interest within known areas of ablated and unablated tissue and the extracted spectra were then used to identify the remaining lesions in the field-of-view. A more detailed description of the region-of-interest based linear unmixing approach and its comparison to principal component analysis can be found in our recent publication [7]. Colors were arbitrarily assigned to each component image (green to ablated tissue and red to unablated tissue) to form a pseudocolor *composite* HSI image. To reveal true spectral differences, the raw spectra from individual region-of-interests (ROIs) were normalized and divided by the Nuance FX spectral sensitivity curve provided by the manufacturer. To obtain dimensionless percent change, the difference between ablated and unablated spectra was divided by the peak intensity value of unablated tissue.

### Gross and histopathology

Immediately after completion of imaging studies, tissue was submerged overnight in 40 mM TTC solution causing unablated muscle to turn red. The tissue was then dissected, photographed and used to measure lesion diameter and depth. For histopathology, samples were fixed in 10% neutral buffered formalin, embedded in paraffin, and sectioned into 4μm slices and processed using Verhoeff-Van Gieson and H&E staining.

### Statistical analysis

Ten human hearts from diseased individuals aged 63±9 (mixed gender) was used for these studies. The total number of RF lesions made on the endocardial surface of left human atria was 93 (79 by non-irrigated and 14 by open-irrigated catheter). To test effects of the blood on HSI outcomes, three rats were used. Two bovine hearts were used to correlate spectral changes with lesion depth. Values are presented as mean ± SEM unless noted otherwise, with Student’s t-test values of p<0.05 considered significant. Raw data and calculations used to make individual figures are included in S1 File.

## Results

### Effect of human left atrial structure on visual appearance of RF lesions

**Fig 1:**
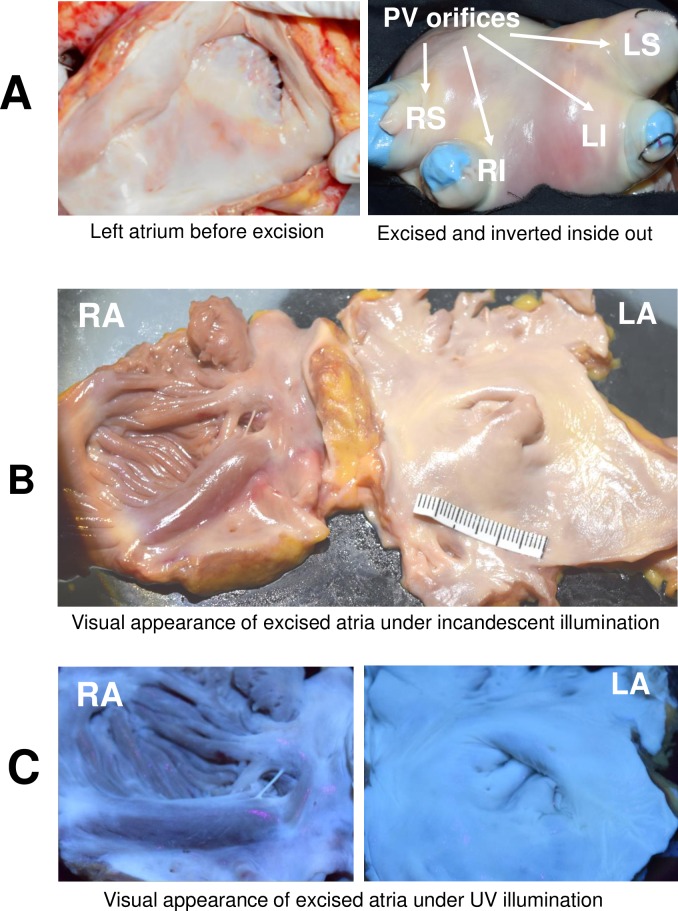
Visual appearance of the endocardial surface of human atria. A. Endocardial surface of left atrium is covered by a thick, heterogeneous layer of collagen including orifices of four pulmonary veins (LI and RI—left and right inferior, LS and RS–left and right superior pulmonary veins). The right panel shows the excised left atrium turned inside-out. B. Appearance of excised human atria under room light. The left atrium appears smoother with a visibly thicker collagen layer. C. Visual appearance of the same tissue under UV illumination.

The endocardial surfaces of both atria are covered by highly reflective and fluorescent layers of collagen and elastin. This collagen layer is particularly abundant in the left atrium including the orifices of four pulmonary veins. It can be readily seen under either white light or UV illumination (Fig 1).

**Fig 2:**
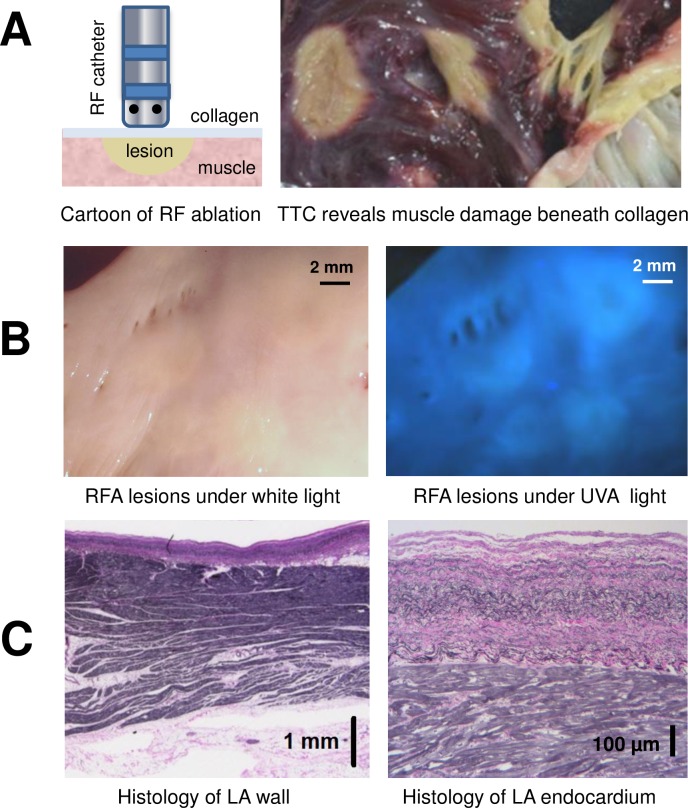
Endocardial collagen layer masks RF-induced damage to atrial muscle below. A. Left: a cartoon of RF catheter ablating atrial endocardial surface. Right: an example of ablated human left atrium after TTC-staining. By peeling off the collagen layer, RF damage to the muscle below can be readily seen (ablated tissue shows as pale areas devoid of red triphenylformazan dye). B. Unstained endocardial surface of human left atrium with multiple RF lesions under either room light or UV illumination. Note the limited contrast between lesion sites and unablated, healthy tissue. C. Histology of left atrial wall shows layers of atrial muscle sandwiched between endocardial collagen layer and epicardial fat. A close-up of endocardial layers reveals interwoven fibers of collagen (pink) and elastin (black).

RF ablation causes the muscle tissue beneath the collagen layer to become necrotic and pale in color. The contrast between ablated and unablated muscle can be further enhanced by post-mortem TTC staining (Fig 2A). For the right atrial surface, RF-induced muscle damage can be seen by the naked eye as its layer of endocardial collagen is relatively thin. In contrast, muscle damage caused by RF ablation in the left atrium is essentially invisible due to thick collagen layer above it (Fig 2B). When histology of left atrial wall was performed at random locations, the average thickness of the endocardial layer of was found to be 0.59 ± 0.03 mm (Fig 2C). Therefore, to visualize ablation lesions in unprocessed human left atrium additional approaches are required.

### HSI and underlying spectral changes enabling lesion visualization

**Fig 3:**
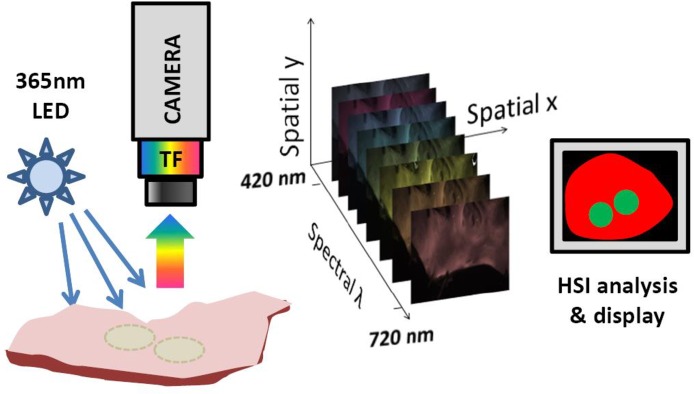
A cartoon of HSI acquisition and processing. Sample is illuminated and fluoresced light is collected thru a tunable filter (TF) coupled to a CCD camera. An HSI hyperstack is a three-dimensional (x,y,λ) dataset comprised of x-y images at different wavelengths.

**Fig 4:**
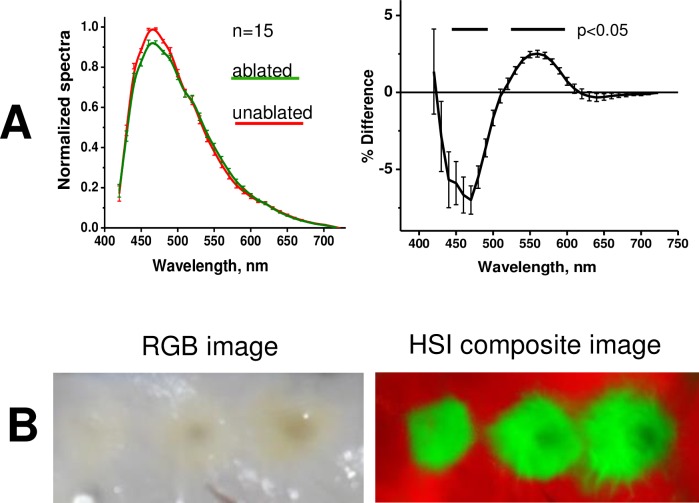
Spectral changes underlying HSI-based RF lesion visualization. A. Autofluorescence spectra from ablated and unablated tissue. The difference between the two is shown on the right. Raw spectra were normalized followed by correction for spectral sensitivity of the tunable filter and quantum efficiency of the CCD and renormalization to maximum values of unablated tissue (details in S1 File). Mean values from 15 RF lesions made on left atrial surface of four human hearts. B. Side-by-side comparison: endocardial left atrial surface with three RF lesions under room light and a composite HSI image of the same tissue

Although RF-induced changes in tissue autofluorescence profile can be too subtle for one’s eyesight, we hypothesized that they are significant enough to reliably delineate the lesions on the endocardial surface of left human atria. HSI is based on acquiring a stack of images from different spectral bands and then using post-acquisition algorithms to classify pixels according to their spectral profiles (Fig 3). The spectral changes that enable HSI-based lesion identification are illustrated in Fig 4. The RF ablation causes an elevation of the normalized spectrum at the wavelengths longer than 520 nm and a decrease at wavelengths shorter than 510 nm (Fig 4A). Both changes are highly significant (p<0.005, profiles derived from 15 lesions made in 4 different human left atria) allowing successful unmixing of pixels from ablated and unablated tissue. An example of a typical appearance of ablated left atrium and the outcome of HSI-based lesion visualization is shown in Fig 4B.

### HSI outlines lesion boundaries with high spatial resolution

**Fig 5:**
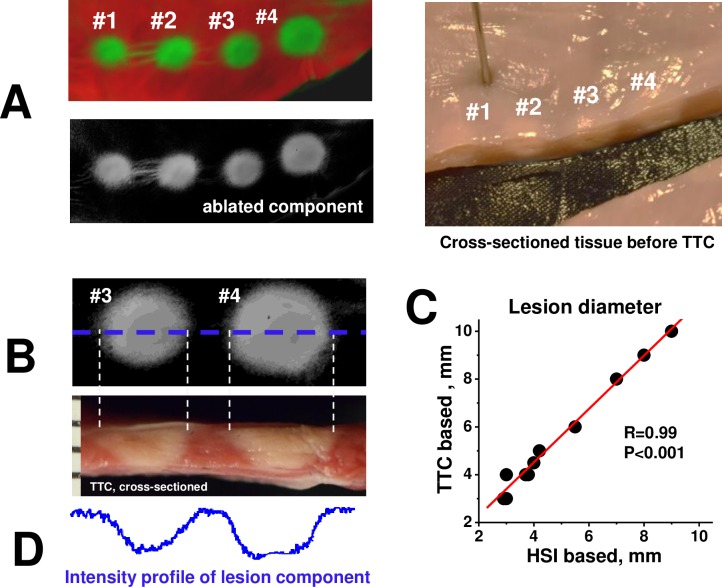
HSI vs histological assessment of lesion size. A. A string of four RF lesions shown as both composite and component HSI image. To the right is the same tissue (before TTC-staining) cross-sectioned through the centers of the lesions. B. Top left panel shows grayscale HSI lesion component image. Bottom left panel is a cross-section of the same lesions after TTC-staining. Dotted lines show measurements of the lesion diameters. C. Graph illustrates the correlation between lesion diameter values derived from histology and HSI lesion component images. D. The intensity plot of HSI lesion component image taken across dotted blue line shown in B.

Histological assessment of lesion dimensions confirmed its strong correlation with HSI outcomes. Fig 5 illustrates how lesion diameter was measured. After an HSI hyperstack was acquired, tissue was cross-sectioned through the center of the lesion (Fig 5A), followed by TTC staining and measurement of the necrotic muscle at the endocardial surface (Fig 5B). The graph in Fig 5C illustrates a near perfect correlation between lesion diameter values obtained from TTC-stained, cross-sectioned samples versus the ones obtained from HSI lesion component images (r = 0.99, p<0.001, 10 lesions from 4 different hearts).

### HSI ability to reveal lesion depth

Knowledge of lesion depth is a key clinical parameter. The potential of autofluorescence-based HSI to reveal lesion depth became evident when intensity profiles derived from lesion component images were compared to their corresponding TTC profiles from transected lesions. A high degree of concordance between HSI lesion component intensity and profile of the lesion depth was clearly evident (Fig 5D).

**Fig 6:**
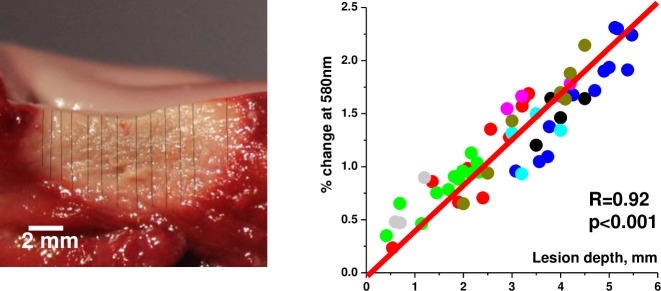
Spectral shift vs lesion depth. This set of experiments was conducted in freshly excised bovine left atria. Several ROIs were selected across each lesion to extract their spectral profiles from HSI hyperstack. Lesion depth at each ROI was then measured. Graph on the right shows the relationship between spectral shift at 580nm and the depth of the lesion (56 ROIs from 8 different lesions, each lesion represented by a different color).

**Fig 7:**
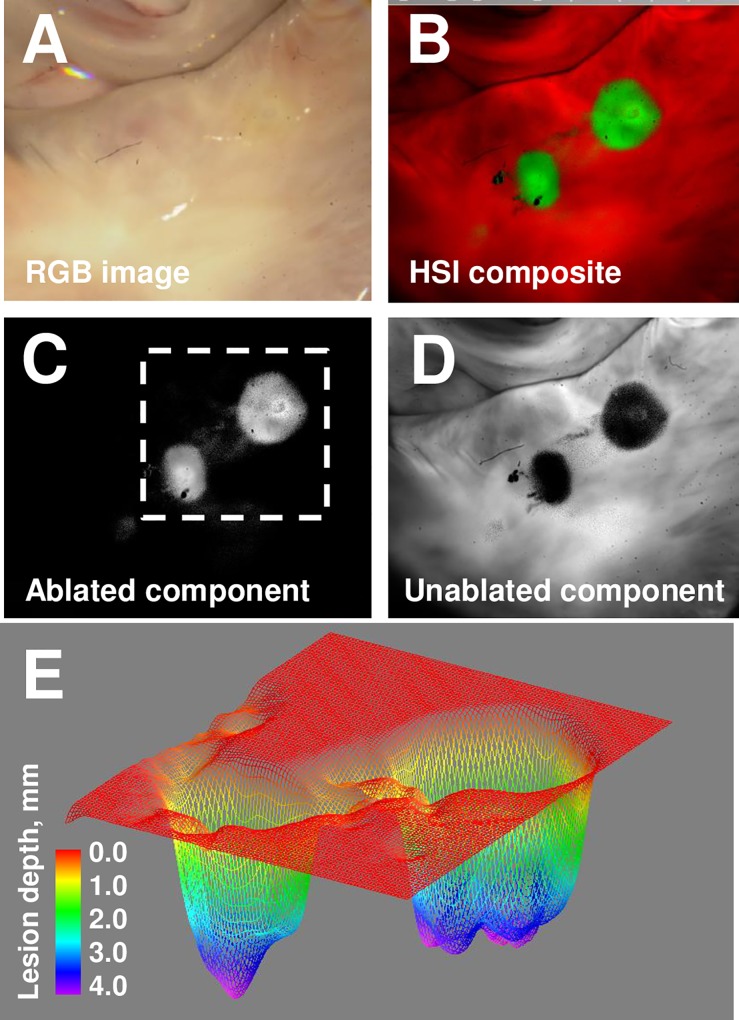
Extracting lesion depth information from surface HSI imaging. A. Appearance of ablated bovine left atrium under room light illumination. B. A composite HSI image of the same area. C. Grayscale lesion component image. D. Grayscale component image of unablated tissue E. Depth reconstruction of lesions from grayscale lesion component image.

In humans, atrial tissue is very thin, therefore over 90% of the lesions we made were transmural upon dissection. To better quantify the relationship between lesion depth and ablation-induced spectral shifts we had to employ a much thicker atrial tissue. Therefore the next set of experiments was conducted using freshly excised bovine left atria. A significant correlation between lesion depth and ablation-induced shift in normalized autofluorescence profiles was observed (Fig 6). The latter can be expressed as a dimensionless percent change at a chosen wavelength. These findings suggest that spectral data extracted from surface HSI imaging can be used to display lesion depth in 3D by calibrating Z-coordinate to pre-acquired spectral shift values (Fig 7).

### Effect of blood on HSI outcomes

**Fig 8:**
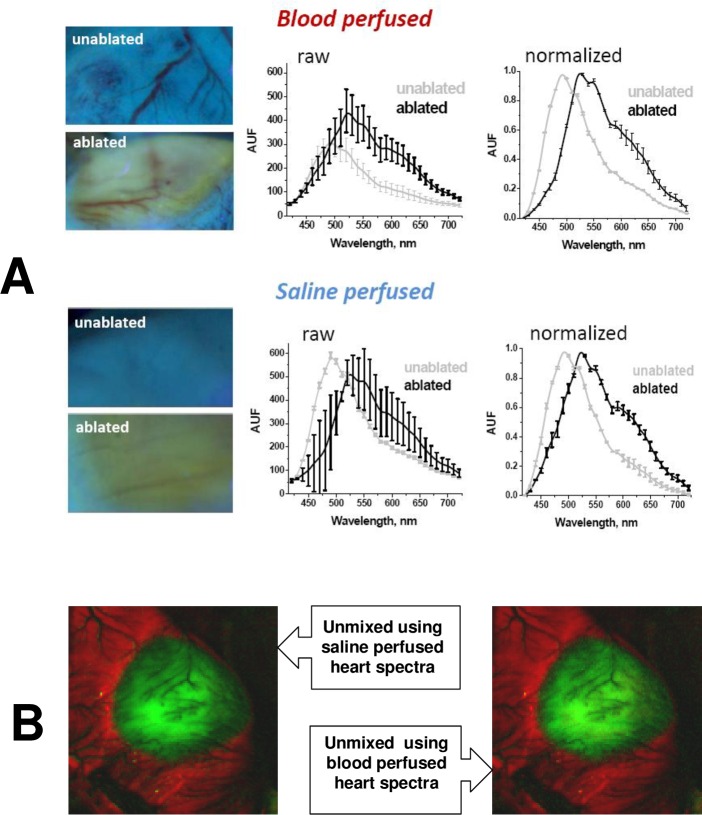
Effect of blood in coronary circulation on HSI outcomes. A.Examples of raw and normalized spectra extracted from HSI hyperstacks before and after ablated rat heart was excised from the animal and perfused with saline to wash out the blood. B.Composite images of an RF-ablated, blood-perfused rat heart showing negligible effect of blood on HSI outcomes. Image on the left was unmixed using pre-acquired spectral library from an excised, saline-perfused heart. Image on the right was unmixed using pre-acquired spectral library from another blood-perfused heart.

Lastly we wanted to confirm that the presence of blood within cardiac muscle will not adversely impact HSI outcomes. To do so, we performed RF ablations in anesthetized, intubated live rats. An animal’s chest cavity was opened, followed by RF ablation of the left ventricle and immediate HSI acquisition. The heart was then excised, perfused with saline and a second HSI hyperstack was acquired. As illustrated in Fig 8A, the presence of blood within the coronary circulation significantly decreased the *amplitude* of the tissue autofluorescence spectrum, particularly for unablated tissue. Yet, when spectra were normalized, there was little difference between spectra from saline-perfused or blood-perfused hearts (graphs on the right). Such similarity in the shapes of normalized spectra enabled us to use the spectra from saline-perfused hearts to successfully unmix an HSI hyperstack acquired in blood-perfused ventricles and vice versa (Fig 8B).

Notably, the error bars in Fig 8A are significantly smaller for the set of the normalized spectra as compared to the raw spectra. This is because the heart surface is not flat and so the amplitude of the raw spectra at each spot depends on its proximity and angle relative to the illumination beam. Spectral normalization greatly minimizes these differences.

## Discussion

When RF ablations are performed on the surface of either ventricles or right atria, the difference in spectral profiles of ablated and unablated tissue is so large that the lesions can be seen with the naked eye without any need for additional approaches[7]. Yet in the left atrium, thick collagen layers yield a highly diffuse reflective endocardial surface, with RF lesions exhibiting negligible contrast under either white light or UV illumination. HSI solves this problem by relying on subtle, yet consistent spectral changes caused by muscle damage beneath the collagen layer.

What are the physical changes that underlie the observed spectral changes? A decrease in normalized spectral profiles which occurs between 440 and 500nm can be ascribed to an acute drop in myocyte NADH levels caused by thermal injury[9]. The elevation in normalized spectral profiles seen between 520 and 600nm can be explained by a marked increase in light scattering at the site of RF ablation caused by protein coagulation[10–13]. This, together with decreased water content[14] causes a larger amount of emitted photons at longer wavelengths to return back to the detector.

One of the main clinical objectives during RF treatment of AF is to consistently apply contiguous lesions in order to electrically isolate the pulmonary veins. Any islands of viable tissue can pose a problem and can lead to AF recurrence. Such viability gaps can be present if lesions are not deep enough or placed too far from each other on the endocardial surface. In our samples the thickness of the LA muscle layer was 2.84 ± 0.61 mm, which is consistent with published data by others[15,16]. Therefore we believe, that in case of human atria, HSI can provide a good estimate of lesion depth using surface illumination, while other techniques, including OCT[17], MRI[18], or ultrasound-based approaches[19] can be of great use in thicker tissues such as the ventricular wall.

Two different mechanisms can be used to explain ability of HSI to reveal lesion depth. Surface UV illumination excites multiple endogenous fluorophores (NADH, flavoproteins, collagen, elastin, lipofuscins) which have broad emission profiles in the visible range. Those visible photons can travel deep into the tissue, yielding changes in autofluorescence profiles within several millimeters of tissue thickness[20]. The second mechanism can be a greater degree of surface damage causing a greater spectral shift at the locations where heat is the highest. The latter, in turn, can cause a deeper lesion.

Clinical application of autofluorescence-based HSI for non-invasive AF therapy guidance requires the development of a percutaneous visualization catheter. Blood is an optically dense fluid. For autofluorescence to be observed it needs to be displaced from the space between the tip of optical fiber and tissue surface. To achieve this, future HSI catheters would need to include an inflatable balloon at the very tip of the catheter. This can be done similarly to other endoscopic catheters[21–23]. Otherwise, the presence of blood within the coronary circulation has minimal impact on the ability of HSI to identify lesions. This is because essentially all visible photons are absorbed by red blood cells when passing through medium and large vessels[24], while the relative volume of red blood cells within capillaries is very small compared to the overall mass of muscle tissue[25,26]. Therefore, while the presence of blood decreases the amplitude of returning visible light spectrum, it has minimal effect on normalized spectral profiles (Fig 8). Since HSI relies on differences in normalized and not absolute spectra to classify the pixels, the presence of blood does not diminish the ability of HSI to identify ablated tissue in vivo.

To implement fiber-based delivery of UV in a percutaneous catheter one needs specialized optics. This has been successfully accomplished by us and others [23,27]. Any adverse effect of UV illumination on cardiac muscle cells is negligible, since the 365nm light used in our experiments is classified as UVA1. The latter delivers much less energy as compared to UVC or UVB because it is in the near visible light range. Our previous studies documented that 365nm illumination does not exert any adverse effects on the amplitude of optical action potentials, heart beating rate, ECG amplitude or NADH levels[28,29]. Notably, in cited studies, ventricular cells were directly exposed to UVA1. For the left atrial tissue, a thick layer of endocardial collagen serves as an additional shield, further minimizing any putative illumination-induced damage to the viable muscle below.

Additional elements of an HSI-based visualization catheter include an imaging fiber optic bundle leading to a tunable filter or filter wheel, interfaced with a camera. The visualization catheter could also be combined with an RF catheter to create a single integrated visualization and ablation catheter. A number of new ablation catheters, recently developed by us and others [22,30] already have most of the above mentioned optical components, including a transparent inflatable balloon to displace the blood, and should be amendable for future HSI-based guidance approach.

Lastly we would like to address concerns about the impact of tissue movement brought by heart contractions or patient breathing on the ability to acquire HSI hyperstacks. The time involved in the acquisition of each hyperstack depends on the number of spectral bands, the intensity of illuminating light and the binning/spatial resolution of the image to be acquired. A smaller number of spectral bands will yield faster acquisition. A higher intensity of illuminating light will also make acquisition faster (i.e., sufficient number of photons will be returned back to the detector sooner). Spatial binning will also shorten acquisition time as more photons are pulled for each pixel. In our settings, typical acquisition lasted about 5 sec yielding 31 spectral bands with 512x512 pixels spatial resolution. This is obviously too slow to acquire a hyperstack from a beating heart, but the main goal of this study was to show HSI’s ability to distinguish ablation lesions in a highly collagenous human atria and not to achieve acquisition rates suitable for in vivo HSI imaging. In the future, the speed of HSI acquisition can be increased in a number of ways. First, only few critical spectral bands can be used to successfully reveal the lesion. Secondly, binning and higher illumination intensity can be used to speed up acquiring the required amount of photons. Alternatively, one can use ECG-gating, in which case HSI acquisition can be timed to ECG signals so multiple images from different spectral bands can be summated across identical parts of the cardiac cycle, similarly to what is routinely used in other types of cardiac imaging. Finally, recent developments of snapshot HSI cameras which have significantly higher acquisition rates, offer yet another path to achieve real-time HSI suitable to for a beating heart.

## Conclusions

We demonstrated, for the first time, the ability of autofluorescence-based HSI to identify RF-induced damage at clinically relevant locations within human left atrium. This imaging approach could help *in vivo* visualization of RF lesions boundaries and reveal interlesional gaps leading to increased procedural success and decreased AF recurrence.

## Supporting Information

S1 File Supporting Information files include raw data and calculations used to make individual figures (ZIP)

## Abbreviations

AF: atrial fibrillation
CCD: charge-coupled device
HIS: hyperspectral imaging
RF: radiofrequency
TTC: 2,3,5-triphenyl-2H-tetrazolium chloride
